# Notch receptor expression in human brain arteriovenous malformations

**DOI:** 10.1111/jcmm.12580

**Published:** 2015-04-03

**Authors:** Sandra Hill-Felberg, Hope Hueizhi Wu, Steven A Toms, Amir R Dehdashti

**Affiliations:** a Department of Neurosurgery, Weis Center for Research, Geisinger Health SystemDanville, PA, USA; b Laboratory Medicine, Geisinger Health SystemDanville, PA, USA; c Department of Neurosurgery, Geisinger Health SystemDanville, PA, USA

**Keywords:** cell signalling, BAVMs, endothelial cells, vascular malformations

## Abstract

The roles of the Notch pathway proteins in normal adult vascular physiology and the pathogenesis of brain arteriovenous malformations are not well-understood. Notch 1 and 4 have been detected in human and mutant mice vascular malformations respectively. Although mutations in the human Notch 3 gene caused a genetic form of vascular stroke and dementia, its role in arteriovenous malformations development has been unknown. In this study, we performed immunohistochemistry screening on tissue microarrays containing eight surgically resected human brain arteriovenous malformations and 10 control surgical epilepsy samples. The tissue microarrays were evaluated for Notch 1–4 expression. We have found that compared to normal brain vascular tissue Notch-3 was dramatically increased in brain arteriovenous malformations. Similarly, Notch 4 labelling was also increased in vascular malformations and was confirmed by western blot analysis. Notch 2 was not detectable in any of the human vessels analysed. Using both immunohistochemistry on microarrays and western blot analysis, we have found that Notch-1 expression was detectable in control vessels, and discovered a significant decrease of Notch 1 expression in vascular malformations. We have demonstrated that Notch 3 and 4, and not Notch 1, were highly increased in human arteriovenous malformations. Our findings suggested that Notch 4, and more importantly, Notch 3, may play a role in the development and pathobiology of human arteriovenous malformations.

## Introduction

Brain arteriovenous malformations (BAVM) are abnormal vascular structures caused by the replacement of normal capillary beds with enlarged and tangled vessels. The lesions are thought to be congenital in origin, and the overwhelming majority of cases are sporadic. These rare lesions can lead to increased blood flow and high pressure in the vessels that can lead to the dilation of feeder arteries and veins. This dilation weakens the vessels making them susceptible to haemorrhage 1.

Brain arteriovenous malformations are often asymptomatic, but symptomatic patients present with headaches, seizures and haemorrhage, potentially leading to potential significant neurological impairment or death. Current treatment modalities, including surgery, endovascular embolization, and/or radiosurgery carry significant risks. Treatment associated risk of morbidity (9%) and mortality (3%) may even outweigh the risk of rupture itself 1. Additionally, some lesions are deemed non-treatable due to their size or location 2. Therefore, the development of alternative treatment strategies with lower morbidities is of paramount interest. Little is known about the underlying biology of BAVMs. The primary factors regulating vessel hierarchy were originally thought to be hemodynamic forces 3. It is now known that genetic signalling contributes to vascular hierarchy, and the Notch pathway has emerged as the critical mediator for the differentiation of arteries and veins 4.

The Notch pathway controls cell fate and influences proliferation, differentiation, and apoptosis during development. Mammalian Notch has four receptor isoforms Notch 1–4 and its mammalian ligands Delta and Serrate 5. Loss of Notch function often leads to reduction of blood vessel diameter, whereas gain of notch function leads to dilation. Notch signaling can also regulate arteriovenous differentiation during embryogenesis, and its role is intertwined with that of the VEGF A pathway 6,7.

The proposed role for Notch signalling in the pathogenesis of vascular malformations was based on abnormalities that resulted from Notch pathway mutations in animal models of vasculogenesis. It has been demonstrated that ephrine B2, a transmembrane ligand, marked arteries but not veins, and the Eph-B4, the ephrine B2 receptor, marked veins and not arteries 4. Notch signalling deficient zebrafish have been shown to lose their expression of Eph B2, where Eph B4, normally associated with veins, was ectopically expressed 8. It has been suggested that Notch signalling was required for proper development of arterial and venous blood vessels and that the major role of Notch signalling was to repress venous differentiation within developing arteries 8. Changes in arteriovenous gene expression profiles of model animals were accompanied by an arteriovenous shunt, a hallmark of BAVMs, between the dorsal aorta and posterior cardinal vein 9.

In mutant mice, endothelial cells, with constitutively active Notch-4, have led to an AVM phenotype and developed cerebral arteriovenous shunting and vessel enlargement by 3 weeks of age. Surprisingly, these malformations were reversible if Notch-4 transgene expression was repressed by doxycycline, demonstrating that the involvement of Notch signalling was not only sufficient to induce but also required to sustain the disease 10.

Notch-1 signalling was found in smooth muscle and endothelial cells of human BAVMs but not in normal control vessels. In addition, Notch-1 ligands, such as Jagged-1 and Delta-like 4 and the downstream effector Hes-1, were also in abundance in human BAVMs 11.

Notch-3 receptors play a key role in the function and survival of vascular smooth muscle cells 12,13. These receptors are thought to be essential for the maintenance of healthy muscle cells in the brain’s arteries. This receptor protein is located on the surface of the muscle cells that surround blood vessels that are specific to arteries. Mutations in other genes of the Notch-3 signalling pathway have been implicated in diseases such as Alagille syndrome, which is caused by a micro deletion of the 20p12 gene, corresponding to the ligand JAG 1. Cerebral autosomal-dominant arteriopathy with subcortical infarcts and leukoencephalopathy is caused by mutations in the Notch-3 gene itself 14.

Although the Notch signalling pathway seems to play a critical role in arteriovenous cell fate determination during vascular development, its function in normal adult vascular physiology and in the pathogenesis of AVMs in humans is still not clearly understood. In this study using tissue microarrays (TMAs), we evaluated the expression of Notch receptor proteins (1–4) in human brain AVMs as compared to control human vessels harvested from patients during epilepsy surgery.

## Materials and methods

### Human AVM samples

Tissue sections, mainly from the white matter, from an 18 patient retrospective cohort that contains eight patients with BAVM resections and 10 control patients with temporal lobe epilepsy, were processed and analysed in this study. A TMA was created from both BAVM and control tissue that was analysed for Notch receptor proteins. A hollow needle was used to remove tissue cores from regions containing blood vessels in the paraffin embedded human samples. Two to four different regions were taken from each BAVM and control sample which were included in the TMA screening for this study. These tissue cores were then inserted in a recipient paraffin block in a precisely spaced array pattern and cut using a Leica RM2235 rotary microtome (Rankin Biomedical Corp., Holly, MI, USA). The TMAs allowed the entire retrospective cohort to be labelled all at once on one single slide. Therefore, all reagent concentrations, temperatures and times were identical for all samples screened (control/BAVM). The patients ranged in age from 2 to 66 years old with a mean age of 42.2 years. All studies involving patients were conducted under protocols approved by the Institutional Review Board at Geisinger Health System.

### Tissue staining

Haematoxylin and Eosin staining (Symphony system; Ventana Medical Systems, Inc., Tucson, AZ, USA) was performed on the formalin fixed TMA sections to identify general vessel features and structures. Verhoeff Van Gieson Elastin Stain Kit (Polysciences Inc., Warrington, PA, USA) was used on the paraffin embedded TMA sections to identify arteries in the samples analysed. CD31 antibody (cat#131M15; Dako, Carpinteria, CA, USA) was used to verify the endothelial cells (data not shown).

### Immunohistochemistry

Immunohistochemical labelling for Notch 1–4 was performed on formalin fixed and paraffin embedded 5 μm TMA sections, according to previously published methods 15. Immunoreactivity was scored based on the observed labelling in the vessels: Negative (−); < 10% (+/−); 25% (+), 50% (++); > 50% (+++).

Slides containing tissue arrays were subjected to heat-induced epitope retrieval (HIER) using either a citrate buffer (pH 6) or EDTA (pH 9). HIER was carried out at 98°C for 20 min. followed by 20 min. of cooling in the same buffer.

Double label immunohistochemistry for the Notch proteins (1, 3, 4) and elastin antibody was accomplished using the EnVision G/2 Doublestain System for Rabbit and/or Mouse (Dako). Since Notch 2 was not detectable in any of our samples, we did not double label using the Notch 2 antibody. The double labelling procedure followed these steps: Dual Endogenous Enzyme Block (5 min.), rabbit anti-Notch 1 (1:200), Notch-3 (1:200), or Notch-4 (1:1000) primary antibodies (45 min.; Abcam, Cambridge, MA, USA), HRP Polymer (15 min.), DAB+ chromogen (3 min.); Doublestain Block (3 min.), elastin primary antibody (35 min.), Rabbit/Mouse LINK (10 min.), AP Polymer (15 min.), Permanent Red chromogen (5–6 min.). The tissue arrays were counterstained with Gill 2 Haematoxylin, dehydrated and coverslipped using Permount mounting media (Electron Microscopy Sciences, Hatfield, PA, USA).

### Western blotting

Fresh white matter tissue samples from four BAVMs and three control epilepsy surgical resections where processed and used for performing western blot analysis for the purposes of verifying our TMA findings. 50% Percoll/50% Minimum Essential Media (MEM; Gibco/Invitrogen, Grand Island, NY, USA) was prepared in a 5 ml tube. The Percoll/Mem mix was spun at 39,200 × g for 1 hr at 4°C. The BAVM tissue was minced and placed in a homogenizer with 4 ml MEM using a mortar and pestle. 2 ml of the homogenized tissue was carefully layered on top of the Percoll gradient and spun at 1700 × g at 4°C for 10 min. The upper and lower layers of the gradient were removed, and the middle layer containing the enriched endothelial cells was collected, and washed with media. It was then centrifuged in an Eppendorf 5415 rotor at 9300 × g for 1 min. at 4°C. The supernatant was discarded, and the pellet was re-suspended in 1× RIPA buffer (Sigma-Aldrich, St. Louis, MO, USA) with a complete protease inhibitor tablet (Roche, Mannheim, Germany) added. The supernatant was sonicated for 5 sec. and frozen at −80°C for Westerns. Protein concentrations were determined using a Pierce Protein assay kit (Thermo Fisher Scientific Inc., Rockford, IL, USA). 20 μg of protein were loaded for each sample on a 4–12% gradient Novex Criterion gel (Invitrogen, Carlsbad, CA, USA). The gels were run at 200 volts for 1½ hrs. The gel was then transferred onto an Immobilion P membrane (Millipore, Billerica, MA, USA) for 1½ hrs at 100 volts, blocked with blotto (Thermo Fisher Scientific Inc.), followed by the primary rabbit anti-Notch 1 (ab8925; 80 kDa), rabbit anti-Notch 3 (ab23426; 244 kDa) or rabbit anti-Notch 4 antibody (ab33163; 543 kDa) overnight. Notch 1–4 antibodies were against the cleaved form of the protein (1:500; 1:1000, 1:100; Abcam). Secondary antibody goat anti-rabbit horseradish peroxidase (Jackson Immunochemicals, West Grove, PA, USA) was added for 1 hr at room temperature. Immunoreactive proteins were detected with Super Signal Western chemiluminescence (Thermo Fisher Scientific, Inc.) substrate and developed on x-ray film (Blue 5 × 7) (Midsci, St. Louis, MO, USA).

## Results

**Figure 1:**
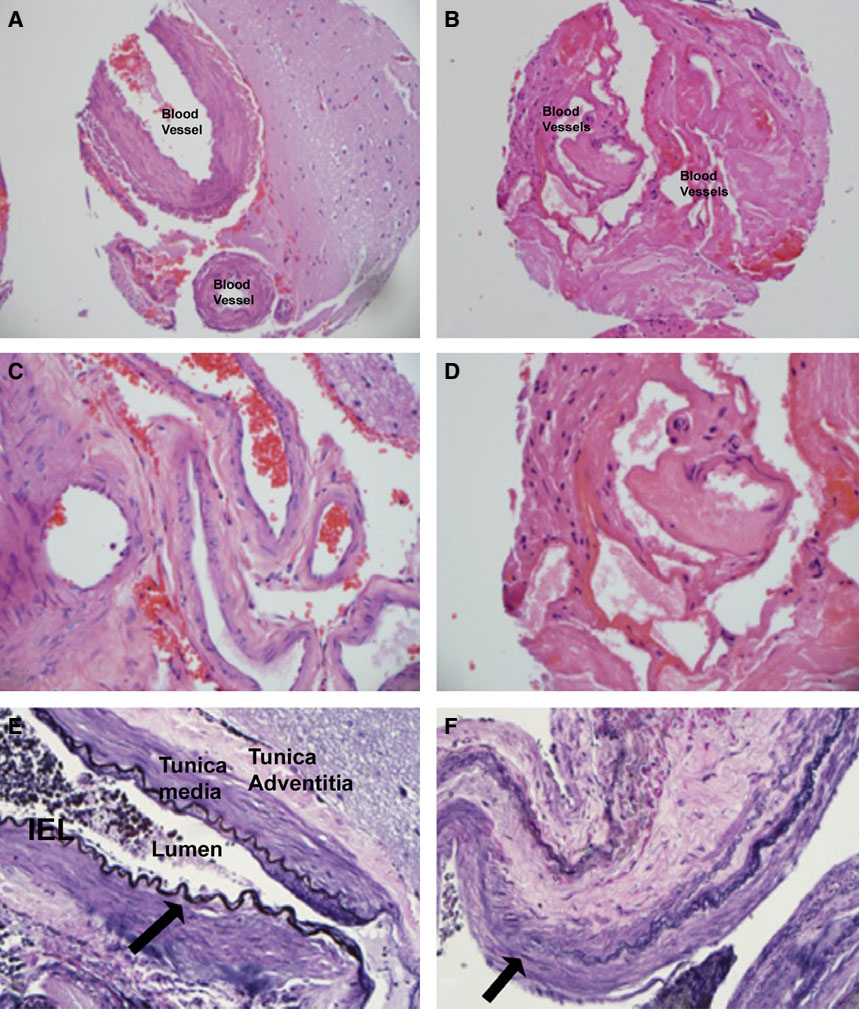
Haematoxylin and Eosin staining of control (A and C) and AVM blood vessels (B and D). Elastin Antibody labelling in control (E) and AVM tissue (F). Arrows show areas of elastin antibody labelling. 200× (A and B), 400× (C–F).

To screen the levels of Notch 1–4 proteins in human BAVMs and control vessels, we performed immunohistochemistry using specific Notch antibodies on a TMA to simultaneously allow labelling and analyses of the entire retrospective cohort. An elastin antibody, as well as a specific elastin stain was used to identify arteries in the tissue samples. Haematoxylin and eosin stain was used to look at the general histology of the vessels analysed in the TMA. Normal artery vessel wall structure could be seen using haematoxylin and eosin staining in blood vessels removed from control epilepsy brains (Fig.1A and C). We were able to identify a defined internal elastic lamina (IEL) labelled intensely with an elastin antibody (Fig.1E, arrow). This layer bordered the inner Tunica Intima containing the flattened endothelial cells. Tunica Media layer contained primarily smooth muscle cells and the Tunica Adventitia layer containing connective tissue.

The BAVM tissue had abnormal artery wall structure when stained with haematoxylin and eosin (Fig.1B and D). We could not identify a consistently defined IEL with the elastin antibody in the BAVM samples (Fig.1F; arrow). The layers appeared disruptive, and the pattern of elastin staining was abnormal (Fig.1F).

**Figure 2:**
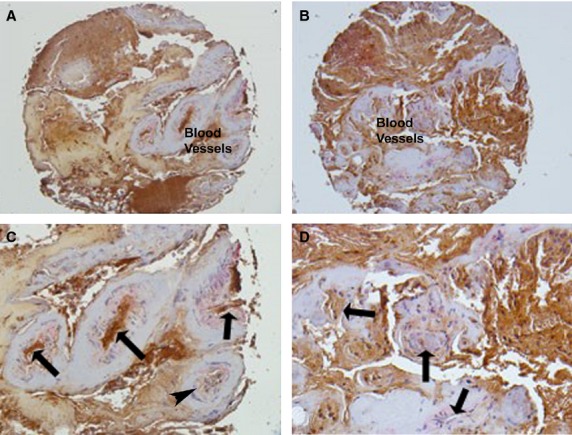
Immunohistochemistry for Notch 1 in control (A and C). Arrows indicate Notch 1 present in the interior wall of the blood vessels of the control sections. Arrow head indicates a blood vessel with minimal Notch 1 labelling. BAVM TMA samples (B and D). Arrows indicate vessels with decrease Notch 1 labelling in BAVMs. 200× (A and B), 400× (C and D).

The Notch 1 antibody that was used in this study recognized the cleaved intracellular form of Notch 1. When this antibody was used on control and BAVM on our TMA, we found that the overall Notch 1 labelling had decreased in the internal artery vessel walls from a score of approximately 25% (+)in control vessels to a score of approximately <10% (+/−) label in the BAVM vessels (Table1). We identified that Notch 1 (Fig.2) was expressed most intensely on the IEL of the vessel wall in control arteries (Fig.2A and C; see arrows). The labelling appeared to be in the same layer as the elastin antibody labelling from Figure1E. An arrow head indicates a blood vessel where there was minimal Notch 1 labelling (Fig.2C). We identified a few of these throughout the tissue but overwhelmingly the vessels were highly Notch 1 positive in the control samples.

**Table 1:** Summary of results on patient vessels labelled with Notch 1-4

DX	Age	Gender	Notch 1	Notch 2	Notch 3	Notch 4
AVM	24	F	+	+/−	++	++
AVM	27	F	+	−	+++	++
AVM	40	M	−	−	+	+/−
AVM	61	M	+	+	+++	+++
AVM	44	F	+/−	−	+	+/−
AVM	42	M	+	−	+++	++
AVM	2	M	−	−	+++	+/−
AVM	66	M	−	−	+	−
Control	39	F	+	−	+	+
Control	51	M	+/−	−	+	+/−
Control	49	F	+	+	+	+
Control	37	F	+/−	−	+	+/−
Control	29	M	+	−	+	+
Control	57	F	++	−	+	+
Control	38	M	++	−	+	+
Control	35	M	+	−	+	+
Control	53	M	+	−	+	+
Control	66	M	++	−	+	+

Negative (−); Minimal staining <10% (+/−); 25% (+), 50% (++); > 50% (+++).

**Figure 3:**
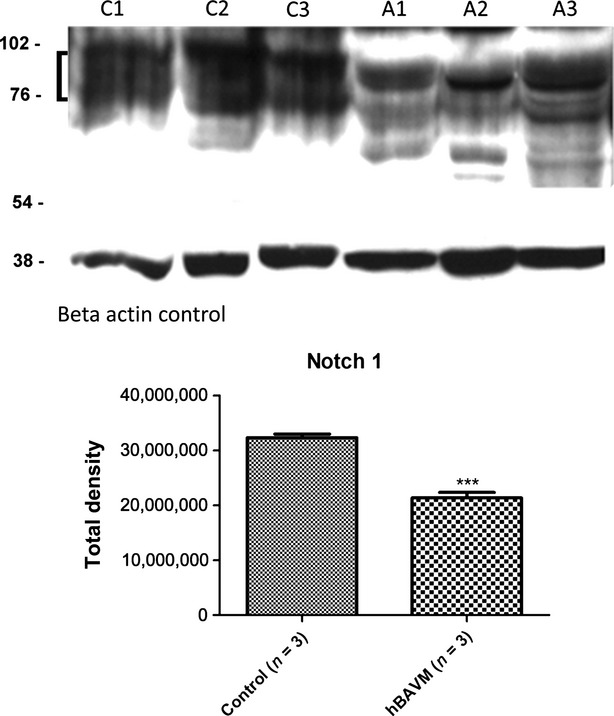
Western Blot for Notch 1 in control (C1–3) and AVM (A1–3) fresh surgical samples. Multiple bands could be identified between 76–150 MW. The bracket indicates the bands that were included in the quantification. Bar graphs represent Total Density after they were normalized with the B-actin internal control antibody. Graphs were made using Graph Pad Prism 5 Software, ***P = 0.0007.

Notch 1 labelling in the BAVM vessels was dramatically decreased and almost absent in the IEL of the vessel wall (Fig.2B and D; see arrows). Because of the lack of staining, it was hard to identify the IEL in some of the vessel walls in BAVM tissue. This was similar to the decrease in the elastin antibody labelling of the IEL from Figure1F. Western blot analysis for Notch 1, on the endothelial cell enriched percoll gradient layer, demonstrated a significant 1.5-fold decrease (***P = 0.0007) in the Notch 1 protein in BAVMs as compared to control protein levels (Fig.3), supporting the TMA findings for Notch 1.

Notch 2 did not appear to label vessels in either control or BAVM tissue that we analysed (Table1).

**Figure 4:**
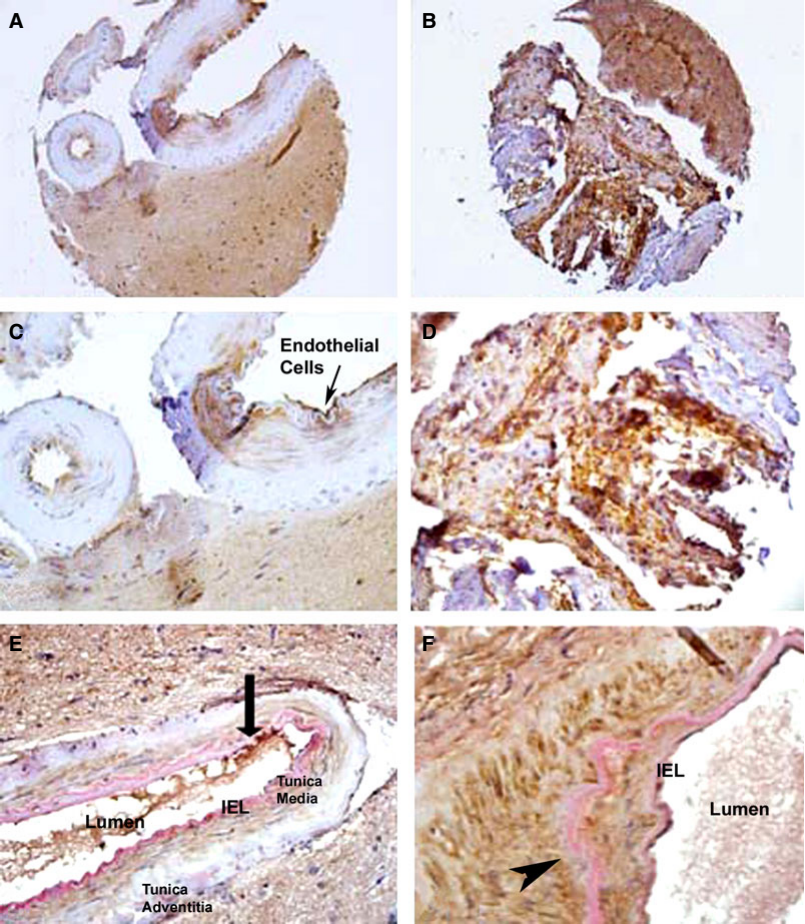
Immunohistochemistry for Notch 4 protein in control (A and C) and co-labelled with elastin (E). Arrow indicates the elastin staining in the IEL of the blood vessel shown. Immunohistochemistry for Notch 4 protein in BAVM tissue (B and D) and co-labelled with elastin (F). Arrow head indicates elastin staining. 200× (A and B), 400× (C–F).

**Figure 5:**
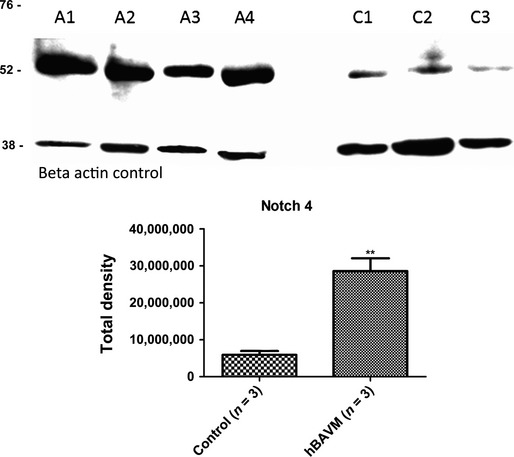
Western Blot for Notch 4 protein in AVM (A1–4) and control (C1–3) fresh surgical samples. A single band at molecular weight 53 could be detected for Notch 4. Bar graphs represent Total Density after they were normalized with the B-actin internal control antibody. Graphs were made using Graph Pad Prism 5 Software, **P = 0.0030.

Overall, Notch 4 had an increase in staining in the BAVM vessels and surrounding tissue (Fig.4B and D) when compared to control epilepsy vessels (Fig.4A and C). We found that the overall labelling intensity did vary among the different BAVM patients analysed. However, age was not a factor with the samples that we analysed. We found a score anywhere from <10% (+/−) to >50% (+++) for Notch 4 labelling, whereas, control arteries generally had the same intensity score of 25% (+) (Table1). Notch 4 labelled the IEL of the blood vessel walls in control vessels (Fig.4A and C). It also appeared to label the endothelial cell layer (Fig.4C). When Notch 4 was combined with elastin staining (pink stain), we confirmed that a portion of Notch 4 was associated with the elastin positive IEL of the artery wall (Fig.4E; see arrow). The majority of the increase in Notch 4 labelling in BAVMs appeared to be due to a diffuse labelling throughout the disrupted vascular tissue (Fig.4B and D). The Notch 4 antibody could be detected on many structures throughout the vessel wall (Fig.4F). The elastin antibody labelling also appeared disrupted (Fig.4F; arrow) similar to the pattern seen with the elastin stain in Figure1F. Western blot analysis for Notch 4 revealed a significant 4.8-fold increase (**P = 0.003) in total Notch 4 protein in the BAVM samples as compared to controls (Fig.5).

**Figure 6:**
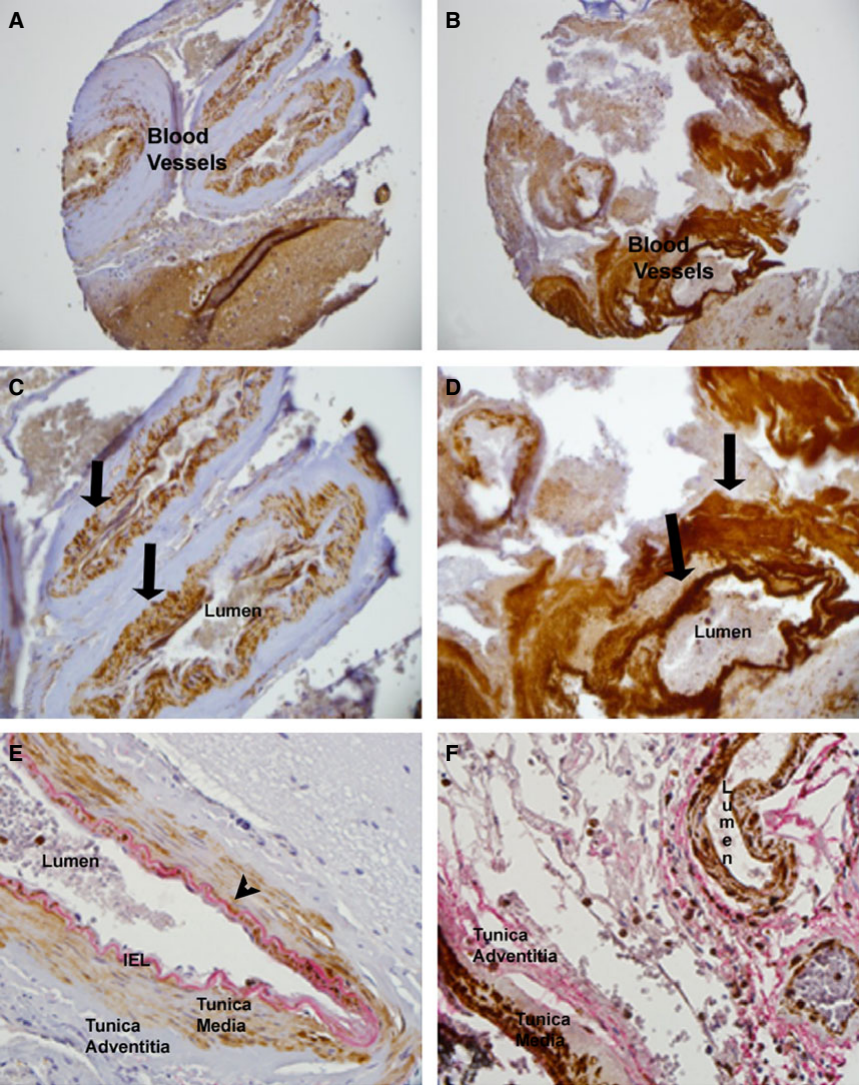
Immunohistochemistry for Notch 3 in control epilepsy (A and C) and co-labelled with elastin (E). BAVM tissue (B and D) and co-labelled with elastin (F). Arrows indicate Notch 3 labelling on blood vessel walls. Arrow head indicates elastin staining in the IEL of control vessels. 200× (A and B), 400× (C–F).

Notch 3, on the other hand, had the most dramatic increase in overall labelling in the BAVM vessel walls. We found vessels in the control tissue that had a score of about 25% (+), whereas in the AVM vessels, we found that most vessels had >50% (+++) labelling (Table1). Notch 3 antibody labelled the IEL and throughout the smooth muscle cell layer of the artery wall (Fig.6A and C), and the intensity of labelling as well as the extent of labelling increased dramatically in BAVM vessels (Fig.6B and D; arrows). The vessels were highly disorganized, and the Notch 3 labelling appeared dramatically increased in both the IEL and the smooth muscle cell layer (Fig.6B and D). We were unable to perform a reliable Western blot on Notch 3 because of the high molecular weight of this receptor, and therefore the inconsistency of running and transferring of this protein. When Notch 3 labelling was combined with elastin staining on control vessels, the Notch 3 receptor labelling could be identified in the elastin positive IEL and the smooth muscle cell tunica media layer of the arteries (Fig.6E; see arrow head). This labelling pattern was completely disrupted in the AVM arteries. The elastin protein appeared to label the connective tissue in the Tunica Adventitia surrounding the IEL and can also be seen in the smooth muscle layer of the Tunica Media where Notch 3 is highly expressed in the BAVMs (Fig.6F; see arrow).

## Discussion

In this study, we found that the overall structural integrity of the arteries in the BAVMs were very disrupted. The pattern of elastin labelling also appeared abnormal in the BAVMs. We found that the active form of Notch-3 was normally present in the IEL and smooth muscle layer of cerebral blood vessel arteries and increased dramatically in five of eight human BAVMs that we analysed. Notch 4 was present on control arteries and increased in half of the TMA BAVM arteries that were analysed. The labelling of Notch 4 had a more diffuse pattern of staining in the BAVMS compared to the other Notch proteins. By western blot analysis, we found a significant 4.8-fold increase in Notch 4 protein in BAVMs over controls. This was not surprising since previously ZhuGe *et al*. showed that Notch 4 expression increased on the vessel wall of human BAVMs 16. However, our results revealed that Notch 4 was not as robust as Notch 3 expression in human BAVMS. The diffuse pattern of Notch 4 labelling that we see is possibly a result of the abnormal architect of the vessel wall and tissue, as well as, some non-specific labelling due to the structural defects of the tissue.

We found that Notch 2 protein was not detectable in both control and BAVM vessels. This was supported by the fact that Notch 2 expression has never been identified on vascular cells 17.

Unlike ZhuGe *et al*., that suggested that Notch 1 was not detectable in control vessels, but had increased in AVMs 11, we found that Notch 1 was detectable on control human brain arteries. There were a few vessels in the TMA with low Notch 1 labelling, but that was more of an exception than a rule. Our western findings verified that BAVMs had a significant decrease in Notch 1 labelling compared to control vessels. Although ZhuGe *et al*., did use a similar Notch 1 antibody in some of their labelling experiments 11, we performed all of our immunohistochemistry labelling on TMAs that gave us the advantage of labelling all of our samples simultaneously. We believe that TMAs along with our supported findings by western blot make a strong argument that Notch 1 labelling in the vessels of BAVMs are significantly reduced overall in the IEL of arteries. We could not determine if Notch 1 expression was reduced because of a decrease in the number of endothelial cells in the IEL of BAVMs or if the cells themselves make less Notch 1 protein. Future studies addressing this issue should be performed.

Together, our findings suggest that some of the Notch proteins may be involved at some stage in the development of BAVMs. Therefore, it could serve as a potential target for development of treatment strategy. Except for surgical resection, endovascular embolization or radiosurgery, there are no other established treatments for BAVMs. These treatments have their own inherent risks and cannot be offered to a substantial subgroup of patients because of excessive treatment risks. Nearly 20% of BAVM patients are left without adequate treatment options.

Even with these findings, we cannot say with certainty if the Notch pathway is the key factor in the development of BAVMs. BAVMs could develop independently from the Notch pathway, and hemodynamic stress caused by high flow arteriovenous shunting may be secondarily activated including the Notch pathway. We know that BAVMs are dynamic lesions with potential for growth, regression and anatomical changes. Even if we cannot interfere with the development of congenital BAVMs, one possible aim for treatment might be to reduce the growth rate or help in the regression of the BAVMs.

The Notch signalling pathway is activated through direct cell-cell interaction with bindings between the Notch ligands in the signalling cells and the receptors on the responding cell. Importance of this signalling is demonstrated by the fact that targeted disruption of Notch ligands in mice results in embryonic lethality with vascular agenesis. It has also been shown that vascular malformations in Notch 4 over-expressing transgenic mice are reversible if expression of an activated Notch 4 transgene is repressed 10.

Considering previous investigations of the role of Notch pathway in BAVMs, our findings suggest that aberrant Notch 3 signalling, in human brain BAVMs, may have a role in the pathogenesis of BAVM. This could open up new potential therapeutic targets for the treatment of BAVMs. Future studies are essential to determine which Notch pathway genes are differently expressed (up-regulated or down-regulated) in BAVMs as compared to normal brain vessel and to determine whether the aberrant phenotype can be reduced by changing the expression of the altered gene.
